# Predicting transcriptional regulatory interactions with artificial neural networks applied to *E. coli *multidrug resistance efflux pumps

**DOI:** 10.1186/1471-2180-8-101

**Published:** 2008-06-19

**Authors:** Diogo FT Veiga, Fábio FR Vicente, Marisa F Nicolás, Ana Tereza R Vasconcelos

**Affiliations:** 1 Laboratório Nacional de Computação Científica, Laboratório de Bioinformática, Av. Getúlio Vargas, 333 Petrópolis, Rio de Janeiro, Brasil

## Abstract

**Background:**

Little is known about bacterial transcriptional regulatory networks (TRNs). In *Escherichia coli*, which is the organism with the largest wet-lab validated TRN, its set of interactions involves only ~50% of the repertoire of transcription factors currently known, and ~25% of its genes. Of those, only a small proportion describes the regulation of processes that are clinically relevant, such as drug resistance mechanisms.

**Results:**

We designed feed-forward (FF) and bi-fan (BF) motif predictors for *E. coli *using multi-layer perceptron artificial neural networks (ANNs). The motif predictors were trained using a large dataset of gene expression data; the collection of motifs was extracted from the E. coli TRN. Each network motif was mapped to a vector of correlations which were computed using the gene expression profile of the elements in the motif. Thus, by combining network structural information with transcriptome data, FF and BF predictors were able to classify with a high precision of 83% and 96%, respectively, and with a high recall of 86% and 97%, respectively. These results were found when motifs were represented using different types of correlations together, i.e., Pearson, Spearman, Kendall, and partial correlation. We then applied the best predictors to hypothesize new regulations for 16 operons involved with multidrug resistance (MDR) efflux pumps, which are considered as a major bacterial mechanism to fight antimicrobial agents. As a result, the motif predictors assigned new transcription factors for these MDR proteins, turning them into high-quality candidates to be experimentally tested.

**Conclusion:**

The motif predictors presented herein can be used to identify novel regulatory interactions by using microarray data. The presentation of an example motif to predictors will make them categorize whether or not the example motif is a BF, or whether or not it is an FF. This approach is useful to find new "pieces" of the TRN, when inspecting the regulation of a small set of operons. Furthermore, it shows that correlations of expression data can be used to discriminate between elements that are arranged in structural motifs and those in random sets of transcripts.

## Background

Unraveling transcriptional regulatory systems is a key step in understanding the regulation of bacterial biological processes, as a whole. In bacteria, transcription factors (TFs) dictate regulation in a great extent because they are directly involved in environmentally-triggered internal and external signals. This is well illustrated by the fact that 3/4 of the transcription factors (TFs) in *E. coli *have been described as exogenous signal sensing [1]; i.e., they are promptly activated/repressed by changes in extracellular conditions. Nevertheless, our present knowledge of the *E. coli *TRN is limited, as only half of the regulators currently known, and only a quarter of the total number of genes, are included in the TRN [2]. Therefore, the use of computational methods can help revealing additional TRNs and contribute to a better understanding of bacterial systems behavior.

As a consequence, several network inference techniques have been introduced to predict regulatory interactions. Initially, motif discovery algorithms and comparative genomic approaches have been widely applied to the prediction of regulatory interactions by identifying binding sites in DNA sequences. Lately, with the increasing use of high-throughput processes for obtaining gene expression data, protein levels, and DNA-protein interactions, network inference techniques started to make use of these data. Identification of causal or dependence relationships in source data rely mostly on static/dynamic Bayesian networks [3-6], graphical Gaussian models [7-9], and relevance networks [10-12].

In general, these graphical models are used to learn causal relationships among genes or proteins, depending on the data available. The results of the analysis is a (un)directed network, in which the links represent regulatory interactions among the entities of the network. Usually, these methods do not use the available information of the organism's TRN as a prior input for the inference procedure. Instead, these methods recover a whole network from scratch, based only on data.

We propose a different view to tackle the problem of inferring regulatory interactions from high-throughput data, which uses pattern identification rather than causal analysis. Over the years, high-quality regulatory interaction data has been collected for *E. coli*. By using a pattern identification technique, this accumulated knowledge can be easily encoded as learning examples, and then a trained model can infer new hypotheses.

As previous studies have shown [13], *E. coli*'s TRN is made up of building blocks known as structural network motifs. Network motifs are clusters of transcription factors and target genes that are linked together in a specific pattern that not appear in random networks. In *E. coli*, the FF and BF arrangement patterns were identified to be these discrete units. These blocks of the network reveal the mechanisms of regulation employed by the bacterium. In the FF, a global TF *X *regulates a function-specific TF *Y*, and both cooperatively regulate an operon. In the BF, TFs *X *and *Y *regulate a pair of operons involved in the same biological process. Also, several motifs bundle together and distribute in functional modules that were found to control several cellular processes [2,14].

We present herein a novel approach for inferring regulatory interactions from transcriptome data tailored for *E. coli*. The method uses the information encoded in the *E. coli'*s TRN in the form of motifs, and benefits from the fact that experimentally validated *E. coli's *network is larger than other organisms' TRNs. In this study we show that in the TRN, FF and BF motifs exhibit a joint-expression pattern that differs from a random collection of regulators and targets.

Having this in mind, we designed ANNs classifiers to infer network motifs in *E. coli*. Those ANNs use the information learned from the collection of FF and BF motifs that appeared in the TRN, as well as from gene expression data, to suggest new motifs. Thus, as FF and BF predictors, these ANNs could be applied for inferring novel regulatory interactions in transcriptome data.

We applied FF and BF predictors to study the regulation of operons that participate in efflux transport systems related to multidrug resistance (MDR) in *E. coli*. Lately, clinically relevant strains of human pathogens exhibiting an MDR phenotype have been identified, including *S. aureus*, *P. aeruginosa*, and *E. coli *[15]. To expel toxic molecules out of its outer membrane, these strains switch on their MDRs pumps. This mechanism may also be responsible for turning these strains resistant to antibiotics. Despite their potential role in turning bacteria resistant to antibiotics, which is a major health problem, little is known about these transport systems. Only 14 (~12%) of *E. coli *TFs have been described to modulate MDR pumps, which is a tiny proportion of the TRN [16]. In addition, recent microarray studies [17-25] that analyzed *E. coli *K-12 grown under anaerobic, aerobic, alkaline, or acid conditions have shown that expression of several MDR pumps depends on a given stress conditions.

We built an expression dataset consisting of 58 Affymetrix chips that contained 95.36% of the bacteria genome and which hybridized with RNA samples from bacteria grown in different stress conditions, such as aerobic knock-out, anaerobic knock-out, and pH changes. Then, motif predictors were applied to investigate the transcriptional control of 16 operons that coded for key transport proteins in MDR pumps. This approach was useful to predict novel TFs that regulate the expression of drug transporters operons and which are involved in responding to those stress conditions.

## Results and Discussion

The design of the ANNs as motif predictors comprised building the learning dataset, and training the networks and model assessment, as detailed below.

### Assembling the Learning Dataset

The learning dataset is the set of examples that is used by the ANNs to adjust the weight for the classification task. Our learning dataset was built with two classes: true motifs examples that were extracted exclusively from the *E. coli *TRN available at RegulonDB version 5.0 [26], and non-motif samples.

**Figure 1:**
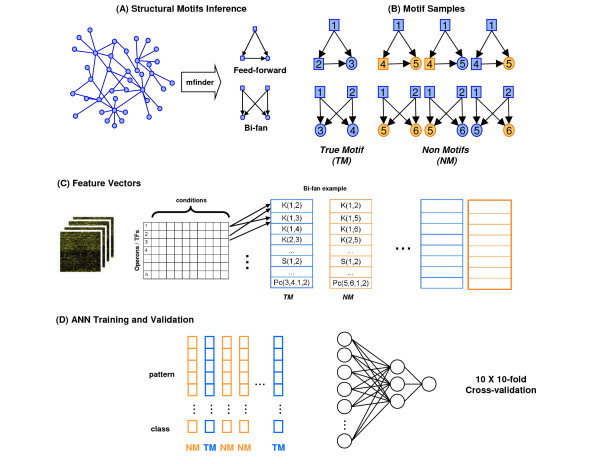
**Steps on designing the artificial neural networks as motif predictors**. (A) Feed-forward and bi-fans instances were extracted from the *E. coli *regulatory network. Square nodes correspond to TFs, and circles to their targets that are either operons or isolated genes. (B) Generic examples of true FF/BF motifs and their counterparts. Non-motif samples were generated by modifying one or more targets of the real motif example, as exemplified in the highlighted orange nodes. (C) Procedures for assembling the feature vectors. Here, there is an example of how the BF motifs *1, 2, 3, 4 *and BF motifs *1, 2, 5, 6 *(illustrated in (B)) are encoded as vectors of correlations. These vectors store the correlations among transcript profiles of motif elements, for all possible pairwise combinations. The k(x, y), s(x, y) and p(x, y), are the Kendall, Spearman and Pearson correlation between x and y, respectively. Also, pc(x, y, z) and pc(x, y, z, t) correspond to the 1^st ^and 2^nd ^order Pearson partial correlation. Therefore, k(1, 2) is the Kendall correlation between the expression profile of TF1 and TF2, k(1,3) is the correlation between TF 1 and its target 3 (an operon or a gene). (D) Learning dataset and the neural network topology used in the study.

Feature vectors were built in three steps. First, we applied Mfinder [27] to find the motifs (Figure 1A). Mfinder identifies interconnecting patterns between nodes (i.e., TFs and operons) that are more frequent than in random networks. Mfinder identified 199 instances of the FF motif and 926 instances of the BF motif in the TRN. These sets of motifs were labelled as the true motif class (TM).

Next, the examples for the non-motif class were generated (NM). For each true motif extracted, a non-motif example was generated by replacing one or more targets of the true motif, randomly chosen among the rest of the operons (Figure 1B). Thus, to create a non-BF motif we could change either the first, or the second, or both targets of the original sample. For instance, consider the BF motif with Crp and Fnr as common activators of *acnA *and *hlyE*. We can modify the targets of these regulators to say, *mtr *and *zwf *because it is known that they do not control these operons. Having this in mind, we assembled a balanced learning dataset with 50% of samples from each class.

The final step involved computing the correlations, and assembling the feature vectors. The expression data was used to compute different types of pairwise correlations, as well as partial correlations, for all operons and TFs in the microarray dataset. These correlation values were later used to assemble the feature vectors. Thus, the correlation values between profiles of motif and non-motifs elements mapped to feature vectors. The example in Figure 1C demonstrates how the 1, 2, 3, and 4 BF motifs, as well as the BF motifs 1, 2, 5, and 6 are represented as feature vectors (see legend of Figure 1C for a detailed description). We assembled six different feature vectors: Pearson correlation only (p), Spearman correlation (s), Kendall correlation (k), partial correlation (pc), Spearman/Kendall/Pearson (skp), and a sixth type containing all previous measures (all). Next, principal component analysis (PCA) was applied in order to reduce the dimensionality of the feature vectors and to optimize the classification task.

### Training ANNs

Using these learning datasets, we trained two types of neural networks, one for predicting FF motifs and one for predicting BF motifs. We trained the predictors using the six feature vectors designs (*p*, *s*, *k*, *pc*, *skp*, and *all*) mentioned earlier in the Assembling Learning Dataset section. All ANNs had the same architecture that included the following: (i) an input layer with a size that depended on the feature vector size after PCA, (ii) a 3-neurons hidden layer, and (iii) an output layer with a single classification neuron (Figure 1D). The over-fitting was avoided using one partition of the data as the validation dataset.

### Validating and Assessing ANNs Performance

The FF and BF predictors were trained and evaluated using a 10 × 10-fold cross-validation, which is a widely adopted approach to evaluate classification algorithms.

**Figure 2:**
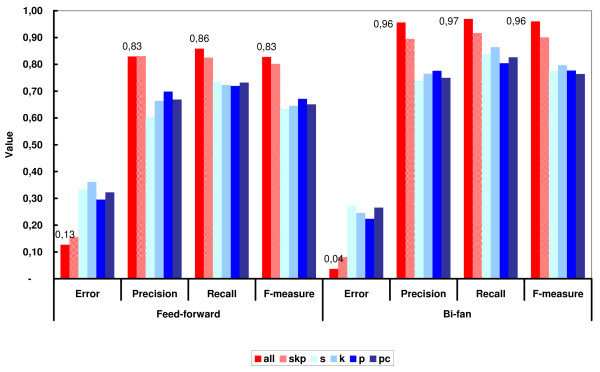
**Classification results of the feed-forward (FF) and bi-fan (BF) neural network predictors**. Error, precision, recall and f-measure rates were measured for the six different feature vector types, namely Pearson (p), Spearman (s), Kendall (k), partial correlation (pc), Spearman/Kendall/Pearson (skp), and another type containing all previous measures (all). Hybrid models (skp and all) outperformed configurations using only one type of correlation (see analysis in the text). All rates represent the average value over the 100 iterations of the 10 × 10-fold cross-validation procedure.

**Figure 3:**
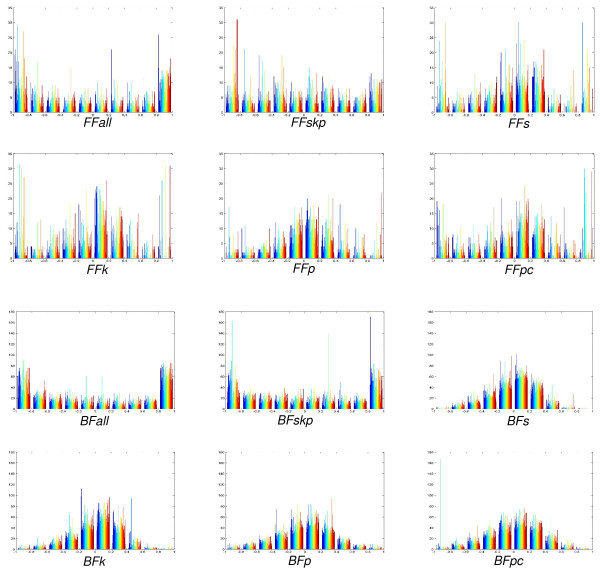
**Output histograms of the feed-forward (FF) and bi-fan (BF) neural network predictors**. Histogram for each type of feature vector, and the output of the classification neuron falls in the range [-1, 1]. The closer to -1, the higher the chances to be distinguished as a motif pattern. ANN classifiers were able to better discriminate whether motif samples were represented using hybrid correlation measures, as shown in *BFall*, *BFskp*, *FFall *and *FFskp *configurations.

This approach consists in splitting the learning dataset in 10 folds of equal size (last fold may have a slight different size). Then, eight folds are used for training (adjusting the weights of the ANN), one fold is used for validation and one fold is used for testing. The validation fold is useful for preventing over-fitting. The testing fold is used to compute the classification statistics, i.e. error, precision, recall (sensitivity) and f-measure (see Methods for definitions). In each cross-validation, all different possibilities of folds for training, testing and validation are chosen. The classification statistics for each cross-validation round are taken as the mean of the 10 possible iterations. These results are shown in Figure 2 (see Additional file 1 for the complete data on classification measures for each cross-validation, including confidence intervals). As shown in Figure 2, for the FF ANNs that use only one type of correlation (*FFp*, *FFs*, *FFk*, *FFpc*), the error rates were similar and around 30%, whereas recalls were nearly 70%. Using hybrid feature vectors, the classification results improved substantially. *FFskp *and *FFall *correctly identified 83% of the samples classified as FF motifs (high precision or positive prediction value) and achieved a high sensitivity (recall) of 83% and 86%, respectively. This indicates that hybrid network models had a low number of both false positives and false negatives. The same was observed for BF ANNs. Error and recall rates for single correlation feature vectors, *BFp*, *BFs*, *BFk *and *BFpc*, were approximately 30% and 80%, respectively. For the mixed correlation networks, *BFskp *and *BFall*, the precision also increased to 88% and 96%, respectively, and the recall to 90% and 97%, respectively. Figure 3 depicts the histograms for the output neuron (class neuron) for each network configuration. The neural network assigned to each sample in the training dataset a real value between -1 (motif) and +1 (non-motif). In this study, configurations using hybrid vectors were able to better separate the two classes because the values were clustered around -1 and 1.

### Regulation of *E. coli *Multidrug Resistance Pumps

The emergence of pathogenic bacterial strains that are resistant to available antimicrobial agents is a major issue in public health. Bacteria strains acquire antibiotic resistance through mutation and selection (vertical evolution), or through exchanging of genetic material (horizontal evolution).

To be protected from the action of drugs, bacteria recruit several efflux pumps that may or may not be specific for a single substrate. These pumps are frequently associated with multidrug resistance (also known as MDR pumps). In prokaryotes, five types of MDR pumps [28,29] have been identified. These are (i) the major facilitator superfamily (MSF), (ii) the ATP-binding cassette superfamily (ABC superfamily), (iii) the small multidrug resistance family (SMR family), (iv) the resistance-nodulation-cell division superfamily (NDR superfamily), and (v) the multidrug and toxic compound extrusion family (MATE) (Table 1). A great interest in how these pumps are regulated has generated substantial research on the regulatory pathways that govern the expression of MDR genes, resulting in the elucidation of transcriptional regulation at both local and global levels. Most efflux pumps known to be under regulatory controls belong to either the MFS or to the RND superfamilies [30] (Table 1). Both families employ the proton- (or sodium-) motive force to energize drug efflux and to trigger regulatory controls. These regulatory controls act by preventing an excessive production of nonspecific cation transport, and by preventing lost of membrane H+ potential, and even cell death. Unlike the MFS and the RND superfamilies, some drug pumps, including the SMR superfamily, do not have their synthesis controlled at the transcriptional level (Table 1).

**Table 1:** Efflux pumps promoting resistance to drugs described in E. coli*.

***(Super)Family***	***Efflux Components***	***Regulator(s)***	***Substrates***
RND	AcrA, AcrB, TolC	AcrR, CRP, Fis, IHF, MarA, MarR, PhoP, Rob, SoxS, SdiA	AC, BL, BS, CM, CV, EB, FA, FQ, ML, NO, OS, RF, SDS, TX
	AcrA, AcrD, TolC	BaeSR, EvgAS	AG, DC, FU, NO
	AcrE, AcrF, TolC	AcrS, Fnr, ArcA	AC, BS, FQ, SDS, TX
	MdtA, MdtBC, TolC	BaeSR	DC, NO
	YhiU/MdtE, YhiV/MdtF, TolC	EvgAS, Ydeo	CV, DC, NO, SDS

MFS	EmrA, EmrB, TolC	EmrR	CCC, NA, TCS, TLM
	EmrK, EmrY, TolC	Fnr, EvgAS, ArcA	CM, TC, SC
	MdfA/Cmr	?	AG, CM, EB, EM, FQ, TC

ABC	YojI, TolC	?	MCJ
	MdlAB	Rob	?
	MsbA	?	EB, EM
	MacA, MacB, TolC	?	EM

MATE	YdhE/NorM	?	AC, FQ, TPP

SMR	EmrE	?	AC, BK, CV, EB, EM, SF, TC, TPP

For each pump was assigned the family, the set of acting proteins, the set of known regulators, and the toxic compounds extruded from the cell.

* As provided in Kumar and Schweizer [16], UniProtKB/Swiss-Prot [47], Ecocyc Database [46] and RegulonDB [26]. Accessions date: Feb 2007.

AC, acriflavine; AG, aminoglycosides; AP, amplicillin; BL, beta-lactams; BS, bile salts; BK, benzalkonium; CB, carbenicillin; CCC, carbonyl cyanide chlorophenylhydrazone; CH, cholate; CM, chloramphenicol; CO, coumestrol; CP, cephalosporins; CT, cefotaximine; CV, crystal violet; DC, deoxycholate; EB, ethidium bromide; EM, erythromycin; FA, fatty acids; FQ, fluoroquinolones; FU, fusidic acid; HL, homoserine lactones; MC, mitomycin; MCJ, microcin J25; ML, macrolides; NA, nalidixic acid; NO, novobiocin; OS, organic solvents; RD, rhodamine; RF, rifampicin; SC, salicylate; SDS, sodium dodecyl sulfate; SF, sulfadiazine; TC, tetracycline; TCS, tetrachlorosalicylanilide; TLM, thiolactomycin; TM, trimethoprim; TPP, tetraphenylphosphonium; TR, triclosan; TX, Triton X-100; ?, unknown or expressed constitutively.

Interestingly, there is increasing evidence that besides conferring resistance to drugs, MDR pumps also play other physiological roles that are required for bacteria survival in their ecological niche [29,31]. In addition, recent studies using microarray have shown that *E. coli *K-12 that are grown under either anaerobic, aerobic, alkanine, or acid conditions express several MDR pumps [17,20,23]. Under oxygen limitations and acid conditions, the expression of the *mdtEF *multidrug resistance efflux increases. When pH increases to a basic condition, the expression of a different pump, namely the acridine efflux pump (*acrAB*), increases. These findings indicate their physiological roles in bacteria survival in natural ecosystems.

So far, only 14 (~12%) of *E. coli *TFs have been described as modulators of MDR pumps, which correspond to a very small proportion of the TRNs. Therefore, a better knowledge about drug transporters and their regulatory networks, along with structural analysis of prominent regulatory proteins, is required for developing drug pump inhibitors. Such pump inhibitors would be helpful to restore bacteria sensitivity to a specific drug or to reduce bacteria ability to colonize and survive in their host.

### Investigating MDR Efflux Pumps Regulation

**Figure 4:**
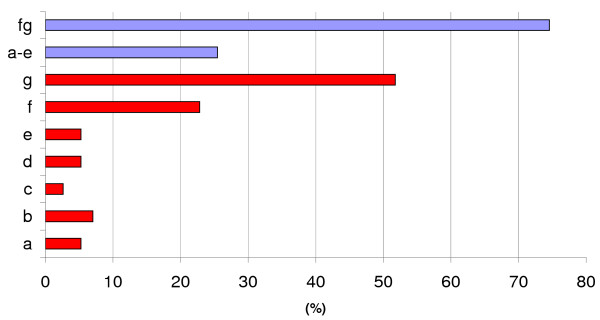
**Distribution of *E. coli *regulators evaluated in this work according to cellular process they control**. TFs appearing in the TRN were annotated in the following categories: (a) local regulator of an MDR efflux pump(s), (b) global regulator of an MDR efflux pump(s), (c) member of an MDR efflux pump regulator family, (d) local or global regulator of non-MDR efflux pumps, (e) regulator of proteins related to efflux pumps or secretion, (f) regulator of an uptake transport system, and (g) regulator of metabolism. Bar labelled a-e represent the summed proportion of the categories (a) to (e) to the whole set of regulators, and are of special interest in this work because they are associated with efflux systems in bacteria.

**Figure 5:**
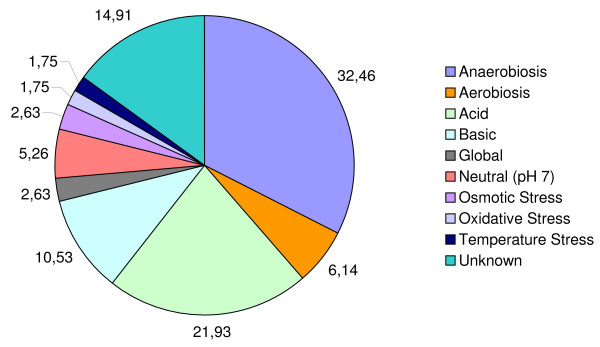
***E. coli *regulators evaluated in this work grouped by stress conditions in which they were described to be modulating their targets**. Evidences have been extracted from a number of genome-wide profiling studies [17-25] and databases [26, 46, 47].

After validation in the learning dataset, we applied the best ANN motif predictors to investigate *E. coli *MDR efflux pumps, with the purpose to find new regulators for proteins involved in these transportation systems in bacteria. To generate the set of testing motif candidates, we first enumerated a target listing of 16 operons (23 genes), corresponding to all operons coding for transporters and accessory proteins as illustrated in Table 1: *acrAB*, *acrD*, *acrEF*, *baeRSmdtABCD*, *cmr*, *emrABR*, *emrErenD*, *emrKY*, *macA*, *macB*, *mdlAB*, *mdtEF*, *mdtK*, *msbA*, *tolC*, and *yojI*. The listing contains a comprehensive review of both well-known and poorly characterized MDR proteins identified so far in *E. coli*. Data was taken from the literature and from organism databases. The next step in our process involved creating motif candidates. For each target operon, large sets of FF and BF candidates were generated making use of the catalog of experimentally characterized inducers and repressors in *E. coli*. This set of 136 TFs (133 operons) selected from the last version of the TRN has been annotated according to their function and into several categories, from *a *to *g*, to facilitates the subsequent analysis of the interactions found by the ANNs (Figure 4, Additional file 5). These categories describe which cellular process(es) a particular regulator is/are controlling and include local and global regulators of MDR efflux pumps (14 instances). TFs directly related to pumping in general (regardless of the type of substract), along with other TFs promoting associated efflux pumps events (categories *a *to *e*), account for 25% of the total; the remaining 75% are regulatory proteins required to uptake transport and metabolism. Additionally, TFs were annotated with respect to a number of stress conditions, in which they regulate the expression of their target(s) (Figure 5, Additional file 5). This search revealed that most regulators showed expression dependence under anaerobiosis (32.46%), acid (21.93%) and basic (10.50%) growth conditions, while regulators able to function under aerobiosis (6.14%) and neutral pH (5.26%) are less frequent.

FF candidates were generated by making all 2-combinations from TFs: $C\begin{array}{c} 133\\ 2 \end{array}$, i.e., ≅ 9·10^3 ^FF testing candidates for each target operon. BF candidates were produced by all 2-combinations from TFs along with operons, except a fixed one: $C\begin{array}{c} 133\\ 2 \end{array}\times 15$, i.e., ≅ 130·10^3 ^BF candidates for every target operon.

**Figure 6:**
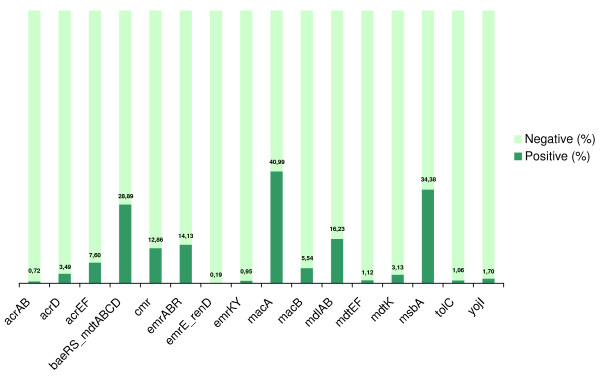
Proportion of samples categorized in the motif class (% TP), for each MDR operon, using *FFall *and a classification threshold of -0.9.

**Figure 7:**
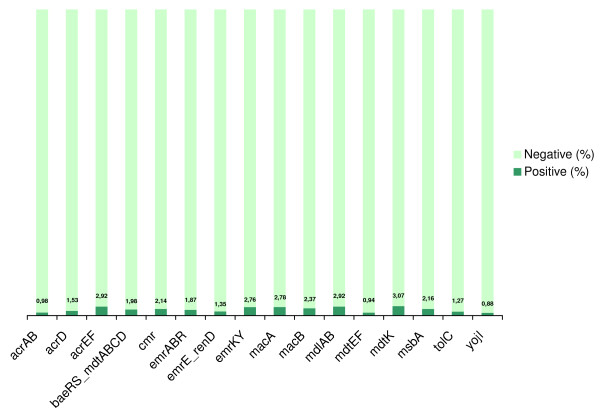
Proportion of samples categorized in the motif class (% TP), for each MDR operon, using *BFall *and a classification threshold of -0.9.

In the last step, we applied the ANN predictors *FFall *and *BFall *to classify the motif hypotheses. Those are the predictors that outperformed other configurations in the curated dataset and which were trained with hybrid feature vectors. Figure 6 gives an overview of the *FFall *results and shows the proportion of candidates classified as true FF motifs using a cutoff of -0.9 for the motif class. It was observed that, for half of the operons, the motif class was assigned to < 4% of the inspected candidates. Figure 7 shows the general functioning of *BFall*. The true positive rates were ≤ 3.07%, for all evaluated MDR operons. This indicates that ANNs are discriminating only a small proportion of the candidates as a motif, which makes it feasible to be analyzed.

**Figure 8:**
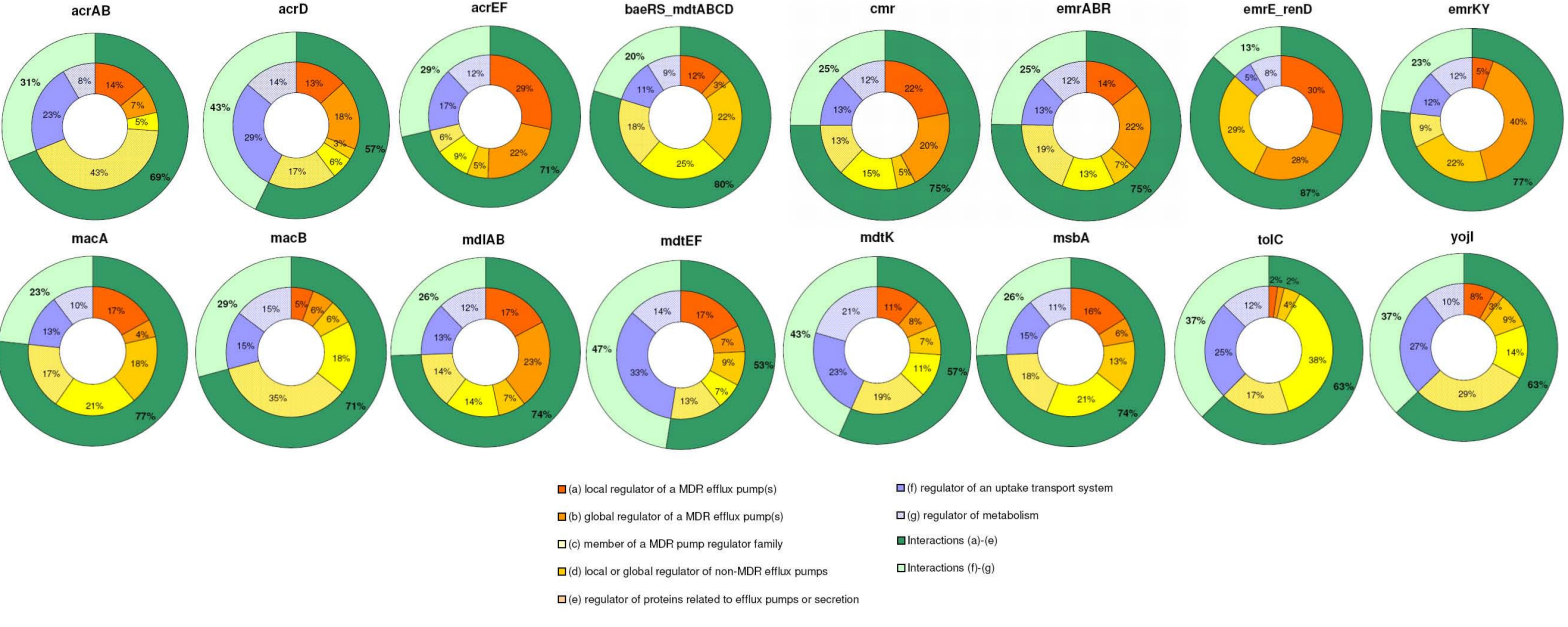
**Outline of the inferred regulatory interactions found employing the FFall predictor**. There are seven types of regulatory interactions, according to the functional classification of the TF. Slice (a)-(e) in the outer donut chart (bold green) represents the set of relationships where a putative binding of the regulator to the promoter region of the operon exists. Refer to text for more details.

**Figure 9:**
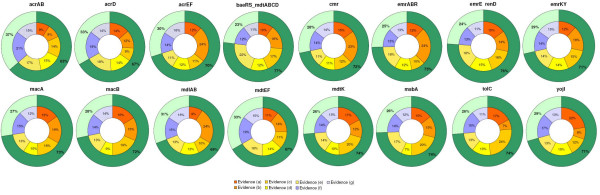
**Outline of the inferred regulatory interactions found employing the BFall predictor**. The color scheme is the same as in the one in Figure 8.

To assess the quality of the predictions, we annotated the set of inferred regulatory interactions using the types of evidence defined for the regulators (see Figure 4 for description of evidences). Regulatory interactions were annotated according to the five types of transcription regulators, namely *a*, *b*, *c*, d, and *e *(Additional file 5). The type *a *transcription regulator was a local regulator of an MDR efflux pump; type *b *was a global regulator of an MDR efflux pump, type *c *was a member of an MDR family of efflux pump regulators, type *d *was either a local or a global regulator of non-MDR efflux pumps, and type *e *was a regulator of proteins related to efflux pumps or secretion. Figure 8 presents the profile of the transcriptional interactions recovered by the *FFall*, for each MDR operon. To construct the chart shown in Figure 8, we considered that any FF motif has two regulatory connections (*TF*_1_-*target*, *TF*_2_-*target*) whereas BF has four, (*TF*_1_-*target*_1_, *TF*_1_-*target*_2_, *TF*_2_-*target*_1_, *TF*_2_-*target*_2_). Each slice in the charts corresponds to the proportion of a given type of interactions *x*, multiplied by a weighting factor 1/*p*(*x*), where *p*(*x*) is the probability of taking a TF of type *x *in the set of all TFs. This weighted proportion is necessary because the number of examples varies between regulators types. For instance, there are many more candidates involving regulators of type *f *than regulators of type *a*. As depicted in the charts, the inner donut shows the percentage for each interaction type, whereas in the outer donut there is the summed *a *to *e *and *f *to *g *percentages. The proportion of inferred a-e interactions is larger in all cases, especially in operons *emrErendD *(87%), and in *baeRSmdtABCD *(80%). Interactions of types *a *to *e *are the most promising ones, because they may represent a DNA-protein interaction between regulator and target, since those *a *to *e *TFs already have been related to regulation of efflux pumps in a direct way. The same was observed about the relationships inferred with *BFall*, where *a *to *e *slices were larger (Figure 9).

In general, this denotes that designed ANNs are preferably selecting MDR regulators, which is biologically consistent. Therefore, we believe that ANNs may infer very plausible hypotheses to be further inspected. Also, there are a considerable number of *f *to *g *interactions recovered by ANNs. Ideally, predictors should identify only *a *to *e *interactions, because *f *to *g *regulators are not responsible for immediately controlling transcription of MDR operons, apparently. These suggest that such interactions between *f *to *g *regulators with the MDR operons were identified because they belong to the same regulatory network of a given stress condition, and their expression profiles correlate likewise real motifs correlate. This also shows that these correlations are far from being the precise representations of molecular binding and they are only an approximate representation for quantifying testable interactions.

### In-depth analysis of selected predictions

We selected some promising hypothesis for a more careful analysis. We first analyzed the inferred BF motif MarA and IscR regulating *acrEB *and *mdlAB *(classification = 0.99112), among the top ten predictions of *acrEB*. Currently it is known that upon reaching a high intracellular level, MarA activates its own transcription and several other genes from the Mar regulon, including *acrAB *(*acrEB *homolog), by binding to the marbox sequence in the promoter region that is upstream of the RNA polymerase site [30]. The marbox is a 20 basepair (bp) asymmetric consensus sequence found in promoters of the *E. coli marA/soxS/rob *regulon(s), where each of the three activators, MarA, SoxS and Rob binds as a monomer [32]. The marbox degenerate consensus was recently updated to: A≠GRGCACRWWNNRYYAAA≠GN (N = any base; R = A/G; W = A/T; Y = C/T) [33]. Although AcrEF is homologous to AcrAB, both efflux pumps can function in combination with TolC and *acrEF *upregulation, which might compensate for an *acrAB *deletion [34]. The *acrEF *operon has not been identified yet as a member of the *mar *regulon and its expression is known to be activated only by Fnr and repressed by AcrS (EnvR) [35]. However, investigation of the promoter of *acrEF *has revealed a sequence of **AAGGCACATA**AA*CA***CAAAA**A that is in the backward orientation and which extends from position 142 to position 161, upstream of the first *acrEF *codon (class I promoter [33]). This sequence matches the *mar *binding site consensus in precisly 14 positions, as defined in the *acrAB *promoter region (underlined), and in 15 out of the 17 most important positions (in bold) according to the most recent marbox consensus [32,33]. Thus, it seems that the *acrEF *promoter region could be a false negative for the marbox consensus sequence containing only two positions, that are inverted compared with marbox *acrAB *(in italic), in disagreement with latest consensus [33]. As a result, we suggest that the *acrEF *promoter region contains a putative *mar/sox/rob *activator binding site at -142 to -161. As such, the feature predicted here, that *MarA *regulates transcription of *acrEF *operon, is feasible for experimental testing. This BF motif also predicts that the regulation of *mdlAB *is *MarA*-dependent. The *mdlA *expression was shown to be enhanced by the MarA homolog Rob, which binds to the *mdlA *marbox promoter DNA [36]. As mentioned above, the highly overlapping *marA/soxS/rob *regulon(s) contain promoters whose expression can be activated by the homologous transcriptional activators MarA, SoxS and Rob. Beacuse *mdlA *is known to be activated by Rob, its expression might also be *marA*-dependent, but that remains to be shown. At last, IscR (regulator 2) was identified by this hypothesis in combination with *mdlA *and *acrEF*. Presently, it is not known if the IscR regulation of the transcription of these targets are done directly or indirectly. In any way, IscR is a member of the Mar/Sox/Rob family of transcriptional regulators and therefore its effect on *acrEF *and *mdlA *can be tested *in vitro*.

Other worth-mentioning case involves the *cmr *(or *mdfA*) operon. The top 20 FF predictions (sorted by classification results) identified EmrR as a regulator of *cmr*, which belongs to the MarR family of transcriptional repressors. EmrR negatively regulates the *emrRAB *operon by directly binding to its regulatory region [37]. This binding is antagonized by some EmrAB pump inducers (CCC, NA and TCS, Table 1) that interact with EmrR and prevent it from binding to this region [37,38]. Besides, there is evidence showing that EmrR might play a regulatory role at the transcriptional level of the expression of several *E. coli *genes [39]. At this point, we suggest that under a given condition of growth and without antibiotics that can antagonize the EmrR function, this regulator acts as a repressor of *emrRAB *and of potential target genes as well. Interestingly, *emrA *shows basic and anaerobic or acid and aerobic dependent expression [17], and thus provide possible evidence that under these conditions some metabolites can act as *emrAB *inducers that antagonize its repression by EmrR. Regarding the several hypotheses associating *emrR *and *mdfA *expressions, we argue that these associations might be representing a repression of EmrR to its targets under acid conditions because the MdfA function is at extreme alkaline pH growth. Both EmrAB and MdfA pumps act as drug/proton antiporters; also MdfA couples proton influx movement to antiport of Na or K ions under extreme alkaline conditions [20]. For example, in an acid/anaerobic growth condition, *emrAB *can be turned off and this repression is mediated directly by EmrR. In a similar condition (acid), this repressor acts directly on the control of other functionally related pumps, such as *mdfA*. We found several other FF high-rated hypotheses and one BF motif that suggest this proposition (see Additional files 2 and 3). Finally, the second regulators that were identified in these predictions could regulate *mdfA *indirectly (positively or negatively), because they have been previously described to have an expression dependence (Additional file 5) on acid (note that the regulator FadR is off when referring to a repressor under a basic condition) neutral (IdnR), stress oxidative (IscR), anaerobiosis (PdhR, Fnr, TorR), and osmotic stress (KdpE).

## Conclusion

ANNs presented here successfully recovered structural motifs available in *E. coli *TRN, reaching high precision and recall rates in this curated dataset. Therefore, the novel TFs predicted to be regulating the expression of MDR pumps proteins are likely candidates to be detected through DNA-protein binding experiments. Enriching the description of FF and BF motifs by using other dependence measures between variables, as mutual information [40,41], could be a future improvement to increase reliability of the classifier. The Matlab source code and all accompanying data are available for downloading at the project website [42].

## Methods

### Assembling of the Expression Dataset

The microarray data comprises 58 *E. coli *Antisense Genome Array chips from Affymetrix with RNA from bacteria grown under different experimental conditions such as aerobic knock-out, anaerobic knock-out, changes of global gene expression during an oxygen shift, and pH changes, was retrieved under the accession code GPL199 from the GEO database [43]. Quantification of probesets and normalization using MAS5 algorithm were performed with the R Bioconductor software package [44]. Probesets annotation was done using the NetAffx tool [45]. In the case of several probesets for the same gene, the mean expression was assigned as the transcript quantity. After computing gene expressions, the last step was to group the genes in operons and compute the expression level of the operon. Considering that an operon is a set of genes that transcribe together, it was reasonable to consider the mRNA levels of the operon as the mean expression of its constitutive genes. The final transcriptome dataset encompasses 784 operons and 2324 individual genes, summing up 4249 genes, or 95.36% of the genome (see Additional file 4).

### ANNs Implementation Details

The entire design of the ANN classifiers was done in Matlab^® ^7.0, using the Neural Network Toolbox v. 4.0.2. Building the learning datasets, including computing of Pearson, Spearman, and Kendall correlations to assemble feature vectors, as well as principal component analysis (PCA), was done using functions of the Statistics Toolbox v. 5.0. The multi-layer perceptron ANNs were designed to have an input layer, a hidden layer with 3 neurons, and an output layer with the classification neuron. The adopted activation function of the perceptrons was the tangent sigmoid. Optimization of the weights was done using the Levenberg-Marquardt algorithm implemented in Matlab (trainlm function). The over-fitting was prevented by using the early stopping criterion. Thus, in each cross-validation, one partition of the data was set apart as validation dataset, and the training stopped when the error in the validation dataset started to increase (defined as the ability of the extrapolation of the network to get worse. Pre-processing of data was performed to scale inputs in the range [-1, 1]. The training parameters learning rate and momentum, were empirically adjusted to 0.5 and 0.1, through minimizing sum squared error (SSE). The size of the the hidden layer was set to 3 based on empirical simulations. The performance of the classification was assessed with the following measures: Error = *FP*/(*TP *+ *FN*), Precision = *TP*/(*TP *+ *FP*), Recall (sensitivity) = *TP*/(*TP *+ *FN*) and F - measure = 2·(Precision·Recall)/(Precision·Recall), where TP is a motif correctly classified, FP is a non-motif classified in the opposite class, and FN is a motif classified in the non-motif group.

## Abbreviations

ABC: ATP-Binding Cassette; ANN(s): Artificial Neural Network(s); BF: Bi-Fan; FF: Feed-Forward; TF(s): Transcription Factor(s); TRN(s): Transcriptional Regulatory Network(s); MATE: Multidrug and Toxic compound Extrusion; MDR: Multidrug Resistance; MFS: Major Facilitator Superfamily; RND: Resistance-Nodulation-cell Division; SMR: Small Multidrug Resistance; PCA: Principal Component Analysis.

## Authors' contributions

DFTV participated in the design of the study, performed neural networks training and evaluation, contributed to biological analysis, and drafted the manuscript; FFRV conceived the original idea, assembled the learning datasets, and helped to draft the manuscript; MFN designed and wrote the biological analysis; and ATRV managed the project and helped to improve the text. All authors read and approved the final manuscript.

## Supplementary Material

Additional file 1 **Error, precision, recall, and f-measure rates for the ANN classifiers in the learning dataset**. Classification statistics were measured using a 10 × 10-fold cross-validation procedure for the six different feature vector types available: Pearson (p), Spearman (s), Kendall (k), partial correlation (pc), Spearman/Kendall/Pearson (skp), and the last type containing all previous measures (all). Confidence intervals were estimated with α = 0.05 (probability = 95%). Click here for file

Additional file 5 Functional classification and stress condition-dependent regulatory patterns for transcriptional regulators investigated in the study*. Each E. coli regulator was annotated according to its functional characteristics such as whether a gene or a controlling target operon, whether a global or local level of transcription, and whether an activator, a repressor, or both. * As found in expression studies [17-25] and *E. coli *databases [26,46,47] (accessions date: Feb 2007). Click here for file

Additional file 2 Complete list of predicted FF motifs involving interactions of types (a)-(e), which were classified by FFall using a threshold of -0.9. Click here for file

Additional file 3 Complete list of predicted BF motifs involving interactions of types (a)-(e), which were classified by BFall using a threshold of -0.9. Click here for file

Additional file 4 Microarray dataset used in this study (see Methods for detail on pre-processing routines used to generate this data). Click here for file
