# Supplementary material for: Predicting transcriptional regulatory interactions with artificial neural networks applied to E. coli multidrug resistance efflux pumps
Source: BMC Microbiol. 2008 Jun 19;8:101. doi: 10.1186/1471-2180-8-101 (PMC2453137; doi:10.1186/1471-2180-8-101)
Supplement: Additional file 5 — Functional classification and stress condition-dependent regulatory patterns for transcriptional regulators investigated in the study*. Each E. coli regulator was annotated according to its functional characteristics such as whether a gene or a controlling target operon, whether a global or local level of transcription, and whether an activator, a repressor, or both. * As found in expression studies [17-25] and E. coli databases [26,46,47] (accessions date: Feb 2007). [file 1471-2180-8-101-S5.doc]

## Table 2 - Functional classification and stress condition-dependent regulatory patterns for transcriptional regulators investigated in the study*.

We assigned every regulator of *E. coli* a particular type according to the operons and/or biochemical process it controls, being that types (a)-(e) are known to be related to efflux pumps, including those directly conferring multidrug resistance -- (a) and (b) types. * As found in expression studies [17-19, 35-40] and *E. coli* databases [21, 41, 42] (accessions date: Feb 2007).

| ***Instances*** | ***Annotation*** | ***Stress Condition*** |
| --- | --- | --- |
| (a) Local regulators of an MDR efflux pump(s) | | |
| EmrR | Repressor, emrRAB operon, MFS MDR pump | Acid |
| EvgA | Activator, acid resistance, osmotic adaptation, and drug resistance | Acid |
| PhoP | Dual regulator, repressor of acrAB operon (acrAB-TolC MDR pump) | Acid |
| Rob | Activator, resistance to antibiotics, organic solvents and heavy metals | Global |
| SdiA | Activator, increase in the expression of the AcrAB MDR pump proteins | Basic |
| TorR | Dual regulator, anaerobic respiration, GO: 0009061, overproduction causes a drug resistance phenotype | Anaerobic |
| (b) Global regulators of an MDR efflux pump(s) | | |
| ArcA | Dual regulator, dye resistance, regulator of EmrKY-TolC pump | Anaerobic |
| Crp | Dual regulator, global regulator in *E. coli* | Global |
| Fis | Dual regulator, initiation of chromosome replication | Global |
| Fnr | Global regulator, anaerobic respiration, GO:0009061 | Anaerobic |
| Ihf | Dual regulator, global regulator in *E. coli* | Anaerobic |
| MarA | Activator, response to multiple antibiotics, GO: 0042493 | Acid |
| MarR | Repressor, response to multiple antibiotics, GO: 0042493 | Anaerobic, acid |
| SoxS | Activator, response to oxidative stress, GO:0006979 | Oxidative |
| (c) Members of an MDR efflux pump regulator family | | |
| BetI | Repressor, member of TetR/AcrR family, response to osmotic stress, GO:0006970 | Osmotic |
| IscR | Dual regulator, member of the Mar/Sox/Rob family of transcriptional regulators | Oxidative |
| SlyA | Activator, member of MarR family of regulators | Acid, anaerobic |
| (d) Local or global regulators of non-MDR efflux pumps | | |
| ArgP | Activator, possibly involved in virulence, arginine transport system | Aerobic |
| CueR | Dual regulator, copper efflux | Anaerobic |
| GadE | Activator, resistance to low pH observed upon overexpression of evgA or ydeO | Acid |
| LexA | Repressor, SOS response, GO:0009432 | Anaerobic |
| YdeO | Activator,response to acid resistance | Acid |
| ZntR | Activator, Zn(II), Cd(II) and Pb(II) transport system | Acid, anaerobic |
| (e) Regulators of proteins related to efflux pumps or secretion | | |
| CsgD | Activator, biofilm formation | Acid |
| EnvY | Activator, genes involved in the porines expression, outer membrane (sensu Gram-negative Bacteria), GO:0009279 | Acid |
| FlhD | Dual regulator, flagellar regulon | Anaerobic |
| LrhA | Dual regulator, flhDC and aerobic respiration, GO: 0009060 | Anaerobic |
| OmpR | Dual regulator, controlling of major outer membrane porin genes, ompC and ompF | Acid |
| QseB | Activator, flagella and motility genes | Anaerobic |
| (f) Regulators of an uptake transport system | | |
| ChbR | Repressor, carbon uptake, carbohydrate catabolism, GO:0016052 | Unknown |
| CytR | Dual regulator, transport (nupC, nupG, and tsx) and the utilization of nucleosides (deoCABD, udp, and cdd) | Unknown |
| Fur | Dual, iron transport | Anaerobic |
| GatR | Repressor, galactitol transport and metabolism | Anaerobic |
| HyfR | Activator, formate uptake transporter (FocB) | Anaerobic |
| KdpE | Activator, high-affinity potassium transport system, response to hyperosmotic stress | Osmotic |
| LacI | Repressor, lactose MFS transport and carbohydrate catabolism, GO:0016052 | Anaerobic, acid |
| Lrp | Dual regulator, high-affinity amino acid transport system | Anaerobic |
| MalT | Activator, uptake and catabolism of malto-oligosaccharides | Basic |
| MetJ | Repressor, L- and D-methionine uptake ABC transport and biosynthesis, GO:0009086 | Acid |
| Mlc | Repressor (DgsA), involved with glucose uptake, GO:0000271 | Neutral pH7, basic |
| NagC | Dual regulator, N-acetyl-D-glucosamine uptake and biosynthesis, GO:0046349 | Basic |
| NanR | Repressor, sialic acid transport and metabolism | Anaerobic |
| NhaR | Activator, cation transport | Acid, anaerobic |
| NikR | Repressor, nickel-specific transport system | Acid, anaerobic |
| PhoB | Dual regulator, sn-glycerol-3-phosphate uptake, phosphorous metabolism, PhoR/PhoB system regulates phosphate regulon | Neutral pH7 |
| SrlR | Repressor, glucitol/sorbitol PTS permease and glucitol utilization, GO: 0016052 | Acid |
| TdcA | Activator, threonine/serine uptake and amino acid catabolism, GO:0009063 | Acid, anaerobic |
| TdcR | Activator, threonine/serine uptake and amino acid catabolism, GO:0009063 | Anaerobic |
| TreR | Repressor, trehalose PTS permease, glucose catabolism, GO:0006006, and response to osmotic stress, GO: 0006970 | Osmotic |
| TrpR | Repressor, tryptophan biosynthesis, GO:0000162 | Anaerobic |
| TyrR | Dual regulator, biosynthesis and transport of aromatic amino acids | Anaerobic, aerobic |
| UhpA | Activator, hexose phosphate MFS transporter | Unknown |
| UxuR | Repressor, carbon uptake | Neutral pH7 |
| XapR | Activator, activates transcription of the xapAB operon | Unknown |
| Zur | Repressor, Zn2+ ABC transporters uptake system | Acid, anaerobic |
| (g) Regulators of metabolism | | |
| Ada | Dual regulator,DNA repair, GO:0006281 | Basic |
| AgaR | Repressor, amino sugar biosynthesis, GO:0046349 | Unknown |
| AlpA | Activator, slpa integrase | Acid, anaerobic |
| AraC | Dual regulator, carbohydrate catabolism, GO:0016052 | Anaerobic |
| ArgR | Dual regulator, arginine biosynthesis, GO:0006526 | Aerobic |
| CadC | Activator, amino acid catabolism, GO:0009063 | Anaerobic |
| CaiF | Activator, amine catabolism, GO:0009310 | Anaerobic |
| CsiR | Repressor, putrescine catabolism, GO:0009447 | Basic, anaerobic |
| CspA | Activator, response to temperature stimulus, GO:0009266 | Temperature |
| CysB | Dual regulator, cysteine biosynthesis, GO:0019344 | Unknown |
| DeoR | Repressor, nucleobase, nucleoside and nucleotide interconversion, GO:0015949 | Acid |
| DnaA | Dual regulator, DNA-dependent DNA replication, GO:0006261 | Unknown |
| DsdC | Activator, amino acid catabolism, GO:0009063 | Acid |
| EbgR | Repressor, carbohydrate catabolism, GO:0016052 | Unknown |
| ExuR | Repressor, hexuronate utilization, GO: 0016052 | Unknown |
| FabR | Repressor, synthesis of unsaturated fatty acids | Anaerobic |
| FadR | Dual regulator, fatty acid metabolism, GO: 0019395 | Basic |
| FhlA | Activator, fermentation, GO:0006113 | Anaerobic |
| FucR | Activator, carbohydrate catabolism, GO:0016052 | Basic |
| GadX | Activator, glutamate dependent acid-resistance (AR) system | Anaerobic, acid |
| GalR | Repressor, carbohydrate catabolism, GO:0016052 | Acid |
| GalS | Repressor, galactose utilization, GO: 0016052 | Acid |
| GcvAR | Repressor complex (GcvA,GcvR), amino acid catabolism, GO:0009063 | Basic |
| GlcC | Dual regulator, glycolate utilization, GO: 0016052 | Unknown |
| GlnG | Dual regulator, glutamine biosynthesis, GO:0006542 | Aerobic |
| GlpR | Repressor, anaerobic respiration, GO:0009061 | Basic |
| GutM | Activator, glucitol utilization, GO: 0016052 | Acid, aerobic |
| HdfR | Repressor, phospholipid biosynthesis, GO:0008654 | Aerobic |
| Hns | Dual regulator, regulation of transcription, DNA-dependent, GO:0006355 | Acid, anaerobic |
| HupA | Dual regulator (HU = hupA+hupB), DNA compaction | Temperature |
| IclR | Repressor, glyoxylate cycle, GO:0006097 | Aerobic, acid, basic |
| IdnR | Dual, carbohydrate catabolism, GO:0016052 | Neutral pH7 |
| IlvY | Dual regulator, isoleucine and valine biosynthesis | Aerobic |
| LctR | Repressor, aerobic respiration, GO:0009060 | Anaerobic |
| LeuO | Activator, leucine biosynthesis, GO:0009098 | Unknown |
| LysR | Dual regulator, lysine biosynthesis, GO:0009089 | Acid |
| MelR | Dual regulator, carbohydrate catabolism, GO:0016052 | Anaerobic, acid |
| MetR | Activator, last step of methionine biosynthesis, GO:0009086 | Basic |
| MhpR | Activator, fatty acid oxidation, GO:0019395 | Anaerobic |
| MngR | Repressor, involved in tricarboxylic acid cycle, GO:0006099 | Anaerobic |
| MtlR | Repressor, involved in mannitol utilization | Anaerobic, acid |
| Nac | Activator, nitrogen compound metabolism, GO:0006807 | Acid |
| NadR | Repressor, NAD biosynthesis, GO:0009435 | Neutral pH 7 |
| NarL | Dual, anaerobic respiration and fermentation | Anaerobic |
| NarP | Dual regulator, anaerobic respiration and fermentation | Anaerobic |
| NorR | Dual regulator, detoxifying nitric oxide (NO) under anaerobic conditions xenobiotic metabolism, GO:0006805 | Anaerobic |
| PaaX | Repressor, carbohydrate catabolism, GO:0016052 | Anaerobic |
| PdhR | Dual regulator, glycolysis, GO:0006096 | Anaerobic |
| PspF | Dual regulator, phage-shock genes | Unknown |
| PurR | Repressor, amino acid catabolism, GO:0009063 | Aerobic |
| RbsR | Repressor, carbohydrate catabolism, GO:0016052 | Basic |
| RcsB | Activator, activiation of colanic acid capsule synthesis (cps) and cell division (ftsZ) genes | Neutral pH 7 |
| RtcR | Activator, ATP hydrolysis and interaction with Sigma54 | Unknown |
| UidR | Repressor, carbohydrate catabolism, GO:0016052 | Unknown |
| UlaR | Repressor, negative regulator of ulaG and ulaABCDEF expression | Unknown |
| XylR | Unknown, xylose utilization, GO:0016052 | Anaerobic |
| YiaJ | Repressor, metabolism of an unknown carbohydrate which generates L-xylulose | Unknown |
| ZraR | Activator, activity of the enzyme hydrogenase 3, two-component regulatory system ZraS/ZraR | Anaerobic |
